# Photodynamic Therapy Using a New Folate Receptor-Targeted Photosensitizer on Peritoneal Ovarian Cancer Cells Induces the Release of Extracellular Vesicles with Immunoactivating Properties

**DOI:** 10.3390/jcm9041185

**Published:** 2020-04-21

**Authors:** Martha Baydoun, Olivier Moralès, Céline Frochot, Colombeau Ludovic, Bertrand Leroux, Elise Thecua, Laurine Ziane, Anne Grabarz, Abhishek Kumar, Clémentine de Schutter, Pierre Collinet, Henri Azais, Serge Mordon, Nadira Delhem

**Affiliations:** 1 Université de Lille, Faculté des Sciences et Technologies, INSERM, CHU-Lille, U1189-ONCO-THAI–Assisted Laser Therapy and Immunotherapy for Oncology, F-59000 Lille, France; martha.baydoun@ibl.cnrs.fr (M.B.); olivier.morales@ibl.cnrs.fr (O.M.); bertrand.leroux@inserm.fr (B.L.); Elise.thecua@inserm.fr (E.T.); laurine.ziane@inserm.fr (L.Z.); anne.grabarz@gmail.com (A.G.); abhishek.kumar@ibl.cnrs.fr (A.K.); clementine.de-schutter@ibl.cnrs.fr (C.d.S.); Pierre.COLLINET@CHRU-LILLE.FR (P.C.); henriazais@gmail.com (H.A.); 2 CNRS UMS 3702, Institut de Biologie de Lille, 59 021 Lille, France; 3 LGRGP, UMR-CNRS 7274, University of Lorraine, 54 001 Nancy, France; celine.frochot@univ-lorraine.fr (C.F.); lcolombeau@gmail.com (C.L.); 4 Unité de Gynécologie-Obstétrique, Hôpital Jeanne de Flandre, 59 000 CHU Lille, France; 5 Service de Chirurgie et Cancérologie Gynécologique et Mammaire, Hôpital de la Pitié-Salpêtrière, AP-HP, 75 013 Paris, France

**Keywords:** photodynamic therapy, folate-coupled photosensitizer, ovarian carcinosis, extracellular vesicles

## Abstract

Often discovered at an advanced stage, ovarian cancer progresses to peritoneal carcinoma, which corresponds to the invasion of the serosa by multiple tumor implants. The current treatment is based on the combination of chemotherapy and tumor cytoreduction surgery. Despite the progress and standardization of surgical techniques combined with effective chemotherapy, post-treatment recurrences affect more than 60% of women in remission. Photodynamic therapy (PDT) has been particularly indicated for the treatment of superficial lesions on large surfaces and appears to be a relevant candidate for the treatment of microscopic intraperitoneal lesions and non-visible lesions. However, the impact of this therapy on immune cells remains unclear. Hence, the objective of this study is to validate the efficacy of a new photosensitizer [pyropheophorbide a-polyethylene glycol-folic acid (PS)] on human ovarian cancer cells and to assess the impact of the secretome of PDT-treated cells on human peripheral blood mononuclear cells (PBMC). We show that PS, upon illumination, can induce cell death of different ovarian tumor cells. Furthermore, PDT using this new PS seems to favor activation of the immune response by inducing the secretion of effective cytokines and inhibiting the pro-inflammatory and immunosuppressive ones, as well as releasing extracellular vesicles (EVs) prone to activating immune cells. Finally, we show that PDT can activate CD4+ and CD8+ T cells, resulting in a potential immunostimulating process. The results of this pilot study therefore indicate that PS-PDT treatment may not only be effective in rapidly and directly destroying target tumor cells but also promote the activation of an effective immune response; notably, by EVs. These data thus open up good prospects for the treatment of micrometastases of intraperitoneal ovarian carcinosis which are currently inoperable.

## 1. Introduction

Ovarian cancer is the fifth most common cancer and ranks sixth among all causes of cancer death among women (65,500 new cases in Europe in 2012 and 42,700 deaths) [1]. This high mortality is due to its uncommon metastatic behavior [2]. In fact, ovarian cancer cells can disseminate from the primary tumor sites and eventually disseminate within the abdominal cavity via the peritoneal fluid [3]. This dissemination allows the malignant cells to bind to the reproductive organs, the bladder, the sigmoid colon, and the omentum, causing an accumulation of abdominal ascites and variable-size implants on the surface of the peritoneum [4]. Two-thirds of peritoneal carcinoma are of gastrointestinal origin, more than 50% of which are of colorectal origin, 20% of gastric origin, and 20% of pancreatic origin. Of the non-digestive cases, more than half are of ovarian origin [5]. Primary cytoreductive surgery followed by different cycles of chemotherapy is considered the standard in the management of newly diagnosed advanced ovarian cancer. However, not all patients are candidates for upfront surgery [6]. Furthermore, optimal debunking surgery has been shown to enhance patient survival, compared to sub-optimal debunking, due to the microscopic spread of inaccessible lesions throughout the abdomen [7]. Although the standard treatment results in a complete response rate of 40–60%, more than 90% of patients relapse within 18 months and ultimately die from the disease. At present, progress in treating ovarian cancer has increased the median survival from 12 months in the 1970s to more than 65 months in the 2000s [8]. However, there is an urgent need to develop effective approaches to treat peritoneal carcinosis of ovarian origin. Photodynamic therapy (PDT) has been used in many areas, including the treatment of cutaneous and pleural diseases [9]. In fact, several clinical trials have detailed the use of this therapy in skin cancer [10] or Glioblastoma [11]. PDT could be a complementary option in combination with surgery and chemotherapy by targeting different mechanisms that can bypass tumor chemoresistance and chemosensitivity mechanisms. More precisely, PDT is a treatment technique based on the combination of photosensitizing molecules (PS), PS-specific excitation wavelength light, and intracellular oxygen, which must be in sufficient quantity [12]. The combination of these three factors will generate cytotoxic reactive oxygen species capable of inducing cancer cell death [13] and, more importantly, to close the tumor-associated vasculature and trigger the host immune system [14]. PDT has demonstrated numerous advantages over conventional cancer treatments. A number of preclinical studies on several animal models have described the pro-inflammatory effect of PDT, capable of inducing an antitumor immune response [15]. In fact, recent evidence suggests that vessel occlusion, ischemia, and direct destruction of tumor cells caused by PDT lead to a significant local inflammatory response [16]. More specifically, the damage induced by the PDT generates various alarm signals that could be detected by the effectors of innate immunity [17]. Furthermore, it is now acknowledged that nascent tumors are eliminated by the immune system, unless the malignant cells are able to escape immune recognition [18]. In fact, the immunosurveillance theory hypothesizes that tumors evolve and eventually progress if anticancer immune responses fail. Consequently, the everlasting efficacy of conventional chemotherapeutics, targeted anticancer agents, and radiotherapies depends on their capacity to reinstate immunosurveillance [19]. Nevertheless, the effect of PDT on the regulation of the immune system remains poorly investigated. Although PDT appears to be more selective than other cancer treatment procedures, the PS used must be much more selective for the tumor, particularly in a more problematic site such as the intraperitoneal cavity. Our previous study [20] described the specificity of a new-generation PS coupled with folate, which is capable of specifically targeting ovarian cancer cells.

Our first objective in this work was to validate the efficacy of a newly patented photosensitizer conjugated to folate on ovarian cancer cells [21]. For this purpose, we evaluated the viability and proliferation rate of two different human ovarian cancer cell lines after PDT treatment. Our second objective was to evaluate the secretion of ovarian cancer cells when subjected to this modality of PDT; in particular, by analyzing the production of cytokines and extracellular vesicles (EVs). Finally, we examined the effect of this secretome and these EVs on human peripheral blood mononuclear cells (PBMC) to define whether PDT treatment is likely to modulate the profile of the effective immune response.

## 2. Materials and Methods

### 2.1. Cell Culture

The ovarian tumor cell lines OVCAR3 and SKOV3 were ordered from the American Type Culture Collection (ATCC). SKOV3 cells were cultured in 50% DMEM medium (4.5 g/L d-glucose, l-Glutamine, Gibco, Thermo Fisher Scientific, Waltham, MA, USA) and 50% F-12 (Ham’s F-12 Nutrient Mix, Gibco, Thermo Fisher Scientific, Waltham, MA, USA) supplemented with 10% heat inactivated fetal calf serum (Gibco, Thermo Fisher Scientific, Waltham, MA, USA). The OVCAR3 cells were cultured in RPMI-1640 medium supplemented and 10% heat-inactivated fetal calf serum (Gibco, Thermo Fisher Scientific, Waltham, MA, USA). Both of the medium cultures were supplemented with 1% penicillin (Gibco, Thermo Fisher Scientific, Waltham, MA, USA). Cells were maintained in an incubator at 37 °C, 5% CO_2_, and 95% humidity. Visualization of cells was done using a Nikon eclipse TS100 microscope.

### 2.2. Isolation of Human Healthy Donor PBMC

Human blood samples were collected from healthy adult donors after obtaining informed consent, in accordance with the approval of the Institutional Review Board at the Biology Institute of Lille (DC-2013-1919). Peripheral Blood Mononuclear cells (PBMC) were isolated from peripheral blood samples by density gradient centrifugation using lymphocyte separation medium (Eurobio, Les Ullis, France) and leucosep 50 mL tubes (Greiner Bio One, Courtaboeuf, France). Obtained purity was over 95%. PBMC were cultured in an ML10 medium made with RPMI 1640 medium supplemented with sodium pyruvate (1 mM), non-essential amino acids MEM 1x, HEPES (25 mM), 2-mercaptoethanol (50 μM), gentamicin (10 μg/mL) (Thermo Fisher Scientific, Waltham, MA, USA), and 10% SVF (Gibco, Thermo Fisher Scientific, Waltham, MA, USA). Cells were activated or not with plated anti-CD3 (1 µg/mL; Miltenyi, Bergisch Gladbach, Germany) and anti-CD28 (100 ng/mL; Clinisciences, MontRouge, France). Cancer cell line supernatant (treated or not), also called conditioned media, was adding to PBMC in 96 round-bottomed plates when stated.

### 2.3. Immune Subpopulation Cells Isolation

The human Natural Killer (NK), B lymphocytes (LB), and naturally occurring regulatory T cells (Treg) were isolated from human PBMC using each cell isolation kit, which were developed for the isolation of untouched human NK (CD56+ CD16+), LB (B Cell Isolation Kit II), and Treg (CD4+ CD25+, Regulatory T Cell Isolation Kit). These were carried out according to the manufacturer’s recommendations (Miltenyi Biotec, Auburn, CA, USA).

### 2.4. Photodynamic Therapy Protocol

Cells cultured in 25 cm^2^, 75 cm^2^, or 96-well plates were suggested to PDT. After 24 h, the medium was replaced by a fresh one containing the new patented PS (for physicochemical properties, see Table 1), consisting of a Folate coupled pyropheophorbide conjugate (patent number WO/2019/016397; 1 mg/100 mL, 0.9 M) [21]. After 24 h, the medium containing the PS was changed and replaced by the normal medium of the cell type after two washing steps with PBS (Gibco, Thermo Fisher Scientific, Waltham, MA, USA). A homogeneous illumination (1 mW/cm^2^) with a 668 nm laser was then performed for 1 h, with a specific device developed by OncoThAI. Thanks to the device, 8 × 75 cm^2^ or 25 cm^2^ or 96-well plates could be illuminated simultaneously and homogeneously, as previously described [22]. After another 24 h, the supernatant was recovered, centrifuged, and then frozen at −20 °C, until further use. Cells were used as collected or directly used for further experiments, as described below. Four groups of cells were used: untreated SKOV3 and OVCAR3 tumor cells (NT), cells treated with PS but without illumination (+ PS), illuminated cells only (+ illu), and cells subjected to the whole PDT process (PDT).

### 2.5. RNA Extraction

Total RNA extraction of the SKOV3, OVCAR3, and PBMC was performed using a RNeasy mini kit (QIAGEN, Courtaboeuf, France), as described by the supplier. Briefly, after lysing the sample, ethanol was added to the lysate in order to grant optimal binding conditions. The lysate was then efficiently washed and loaded onto the RNeasy silica membrane for RNA binding; all contaminants were hence discharged. The water-eluted RNA solutions were then subjected to evaluation of their concentration and purity using a Nanodrop 2000c spectrophotometer with the Nanodrop 2000/2000c v.1.6.198 software (ThermoFisher Scientific, Waltham, MA, USA). In order to be considered sufficiently pure, the minimum acceptable values of the specific absorbance ratios should be as follows: 260/280 ≥ 1.8; 260/230 ≥ 1.6. Pure, concentrated RNA solutions were stored at −80 °C until further use.

### 2.6. Retro-Transcription (RT) and Quantitative PCR

The Superscript^TM^ II Transcriptase Reverse Kit was used for RT (Gibco, Thermo Fisher Scientific, Waltham, MA, USA). Reverse transcription was performed using 1 µg of total RNA. The RT-PCR reactions were performed, for selected genes (Table 2), according to the manufacturer’s instructions using a 2X MESA GREEN qPCR MasterMix Plus for SYBR 258 Assay (Eurogentech, Seraing, BELGIUM), a 96-well qPCR plate (Sarstedt, Nümbrecht, Germany), an optical seal (Dutcher, Brumath, France), and the Mx3005PTM sequence detection system (Agilent technologies, Santa Clara, CA, USA.). In each reaction, 10 ng of reverse transcripted RNA (based on initial RNA concentration) was used. All primers were used at 400 nM in a 20 µL reaction. Quantitative analysis was carried out based on the cycle threshold (Ct) value for each well and calculated using the MxPro software (Agilent technologies, Santa Clara, CA, USA). The results were normalized by three housekeeping (HKG) genes: 18S, GAPDH, and HPRT (Table 2) and data are represented as fold differences by the 2^−ΔΔCt^ method [23], where ΔCt = Ct target gene – Ct HKG.

### 2.7. Viability Assays

SKOV3 and OVCAR3 ovarian cancer cell lines or PBMC was assessed by a bioluminescence based viability assay (CellTiter-Glo^®^, Promega, Madison, WI, USA). Briefly, 2000 cancer cells or 1,000,000 PBMC were seeded per well in a 96-well white-walled and clear-bottomed plate (Corning, Amsterdam, The Netherlands) per time point and then subjected to treatments. Cancer cells were treated or not with PS, light, or both. PBMC were activated as previously mentioned with plate bound anti-CD3 and anti-CD28 or not, and subjected to the supernatant of treated ovarian cancer cells. Then, 24 h, 48 h, 72 h, and 120 h post-treatment, 100 µL/well of the Celltiter-Glo mix was added at room temperature for 10 min and protected from light upon manufacturers’ instructions. The bioluminescence was then read using a luminometer under the Microwin software v4.41 (Centro LB960, Berthold Technologies, Bad Wildbad, Germany). Results are presented as the mean of triplicate wells of three independent experiments and expressed in percentage, according to the NT control (100%).

### 2.8. Proliferation Test

PBMC (10^6^/mL final concentration) were seeded in a 96-well round bottom tissue culture plate (Corning, Amsterdam, The Netherlands), activated using anti-CD3 and anti-CD28 monoclonal antibody, as described earlier, and cultured with different conditioned media or isolated extracellular vesicles in order to explore their effects on the activity of PBMC. Proliferation was measured after the addition of [methyl-3H]-thymidine (1 Ci/well; PerkinElmer, Courtaboeuf, France) for the last 18 h of each time point. At the end of each time point, cultured cells were harvested on a glass fiber filter (PerkinElmer, Courtaboeuf, France) using a Tomtec harvester (Wallac, Turku, Finland). The filter was then sealed in a sample bag (PerkinElmer, Courtaboeuf, France) after drying and addition of scintillation liquid (Beckman Coulter, Brea, CA, USA). Radioactive thymidine, incorporated into replicated cellular DNA by proliferative cells, was detected by scintillation counting using a 1450 Trilux β-counter (Wallac, Turku, Finland). Each proliferation assay was carried out in triplicate and estimated in count per minute (cpm) or, when stated, the results were normalized compared with non-treatment conditions to obtain the relative proliferation. All data are presented as the mean values and standard error of at least three independent experiments.

### 2.9. Flow Cytometry

FOLR1 receptor expression was analyzed by cytometry on the OVCAR3 and SKOV3 cell lines. We used an anti-FOLR1-PE (BioLegend, San Diego, CA, USA) and its IgG2a-PE isotype control (Miltenyi Biotec, Bergisch Gladbach, Germany) to determine the expression of the FOLR1 receptor. A total of 10^5^ cells were taken up in a volume of 200 μL of PBS-/- and the fragment crystallizable receptors (FCR) were blocked with FCR blocking reagent (Miltenyi Biotec, Bergisch Gladbach, Germany) for 15 min at 4 °C. They were then incubated for 15 min at 4 °C in the dark with 2 μL of each antibody. The labelled cells were filled up with 300 μL of PBS-/-.

PBMC were stained using adequate monoclonal antibodies (Table 3) after Fc-R blocking treatment, upon manufacturer’s instructions (Miltenyi Biotech, Bergisch Gladbach, Germany). Samples were acquired using a FACS Canto II flow cytometer powered by the BD FACSDiva software version 8.0.1 (BD Biosciences, Franklin Lake, NJ, USA) and analyzed using the Flow Jo software 10.0.7 (Tree Star Inc., Ashland, OR, USA). Results are expressed as normalized values compared to our control condition.

### 2.10. ELISA

Cytokine detection was carried out on the supernatants of ovarian cancer cells treated or not with PDT: supernatant of untreated cells (NT), Illuminated Cells (ILL), in contact with PS (PS), and cells subjected to PDT (PDT). Supernatants of all cell cultures were harvested and kept at −80 °C until their use for cytokine assays. Cytokine secretions of Interleukin (IL)-6, Transforming Growth Factor (TGF)-β1, IL2, and Interferon (IFN)-γ were determined by the Sandwich ELISA (Enzyme-Linked ImmunoSorbent Assay) method. Briefly, purified primary antibodies were coated overnight at 4 °C in flat-bottomed 96-well maxisorp plates (NUNC, Thermo Fisher Scientific, Waltham, MA, USA) before incubation with samples. The corresponding biotinylated antibodies were added for protein detection, after several steps of non-specific site blocking, sample deposition (overnight at 4 °C), and adequate washing (PBS-Tween 0.05%). The reaction was amplified with Streptavidine-peroxydase (Interchim, Montluçon FRANCE). Cytokine concentrations were finally highlighted with the addition of OPD (10 mg/mL, Sigma-Aldrich, St. Louis, MO, USA). After color development, the plates were read using a Multiskan spectrophotometer at 492 nm. The purified and biotinylated antibodies used were as follows: mouse anti-human IL2, rat anti-human IL6, rat anti-human TGFβ1, and mouse anti-IFN-γ (all from BD Pharmingen^TM^, San Jose, CA, USA). Results are expressed in pg/mL as the mean of triplicate wells after subtracting background values.

### 2.11. Extracellular Vesicles Isolation

SKOV3 extracellular vesicles (EVs) were isolated from in vitro conditioned culture media. Isolation of EVs from the supernatants of non-treated, photosensitizer-only, illuminated-only, or PDT treated cells was carried out by differential centrifugation and flotation on a D_2_0/sucrose cushion, as previously reported [24]. Isolated EVs were then diluted at 1:100 and total protein concentration was quantified according to the manufacturers’ instructions (Biorad, Hercules, CA, USA), based on a Bradford dye-binding method, and read at 595 nm on an spectrophotometer powered by the Ascent™ Software v2.06 (Multiskan RC Thermo Labsystems, Thermo Fisher Scientific, Waltham, MA, USA). EVs were cryopreserved after slow freezing in a frozen container (Mr. Frosty, Nalgene, Thermo Scientific, Waltham, MA, USA) at −80 °C until further use and then added to cell culture at different concentrations, in triplicate assays.

### 2.12. Statistical Analysis

Results are given as the mean of triplicates of at least three independent experiments. Data were analyzed using the Prism 6.0 software (Graph Pad Software Inc., San Diego, CA, USA). All quoted p-values are two-sided, with *p* ≤ 0.05 (*), *p* ≤ 0.001 (**), *p* ≤ 0.0001 (***), and *p* ≤ 0.00001 (****) being considered statistically significant for the first and highly significant for the others.

## 3. Results

### 3.1. Validation of the Efficacy of the PS

#### 3.1.1. PS Targeting Ability: Folate Receptor Gene Expression

**Figure 1:**
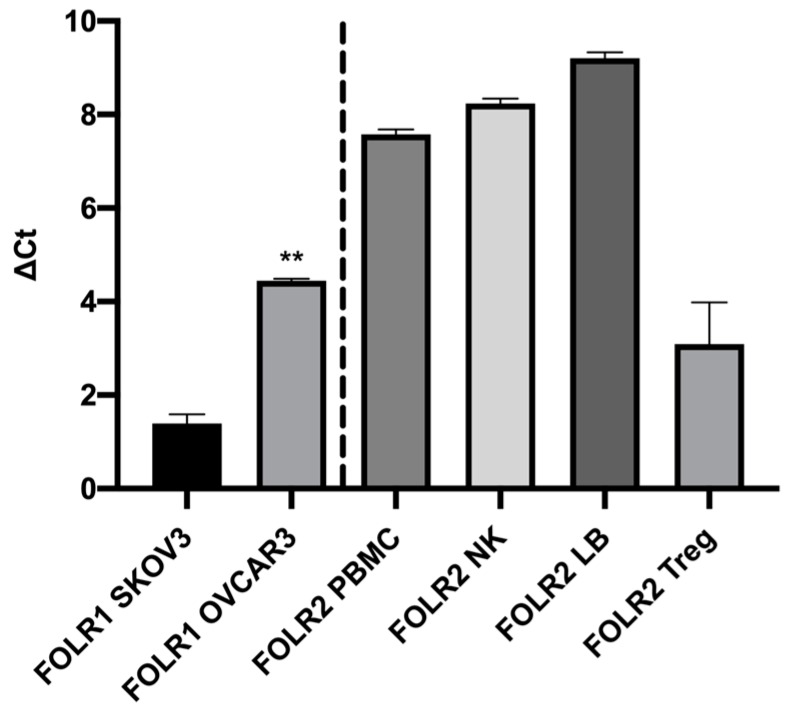
RT-QPCR analysis of FOLR1 and FOLR2 gene expression by ovarian tumor cells and immune cells. FOLR1: Folate Receptor 1, FOLR2: Folate Receptor 2, PBMC: Peripheral blood mononuclear cells, NK: Natural Killer; LB: Lymphocyte B, Treg: Regulatory T Lymphocyte. ΔCt = Ct target gene − Ct HKG. Rank-sum Mann–Whitney statistical test was performed, all quoted *p*-values are two-sided, *p* ≤ 0.001 (**) being considered statistically significant for the first and highly significant for the others.

**Figure 2:**
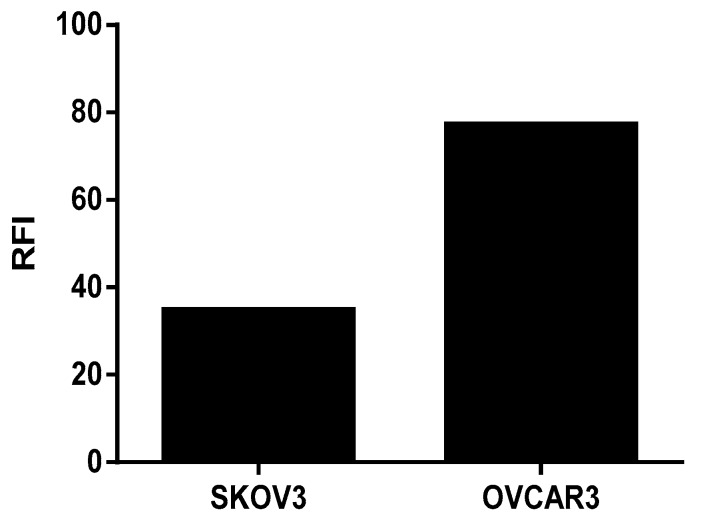
Membrane protein expression of FOLR1 in Ovarian Cancer cell lines using Flow Cytometry and analyzing by the FlowJo Software. Fluorescence intensity representation (RFI).

The transcriptomic analysis shows that the human ovarian tumor cells SKOV3 and OVCAR3 expressed the FOLR1 isoform and that the different isolated immune cells expressed the FOLR2 isoform (Figure 1). In addition, the FOLR1 isoform was more expressed in the OVCAR3 cell line, compared with SKOV3 cells, with a statistically significant difference (*p* < 0.05). This observation was correlated with protein expression level, insofar as we highlighted a more important membranous protein expression of FOLR1 in OVCAR3 than in SKOV3 cell lines (Figure 2). 

#### 3.1.2. PDT Efficacy: Evaluation of SKOV3 and OVCAR3 Shape and Viability

**Figure 3:**
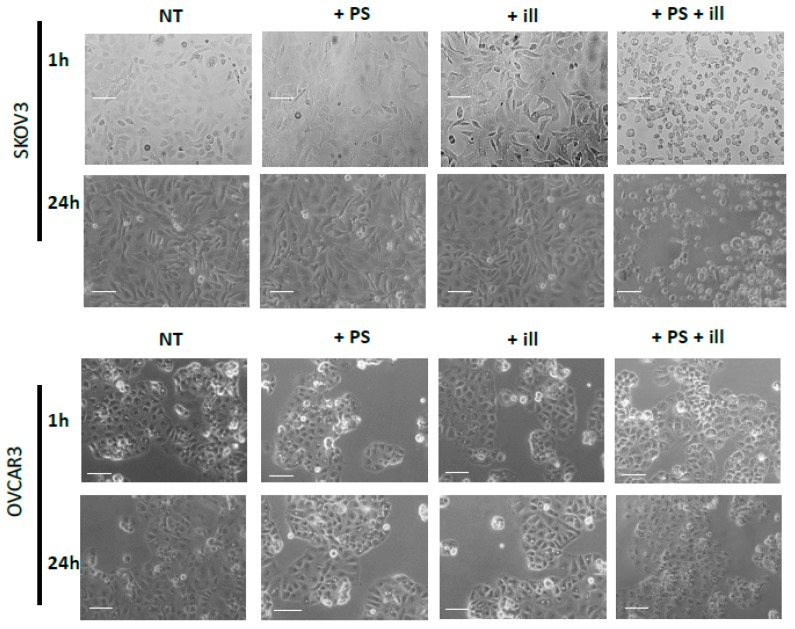
Phase Contrast Image-Based monitoring of OVCAR3 and SKOV3. Morphological aspects of SKOV3 and OVCAR3 tumor cells in different conditions after 1 h (upper lane) and 24 h (lower lane) post treatment. NT: non-treated, +PS: Photosensitizer only, +ill: illumination only; +PS +ill: PDT (illumination in the presence of PS). Bar = 10 µm.

**Figure 4:**
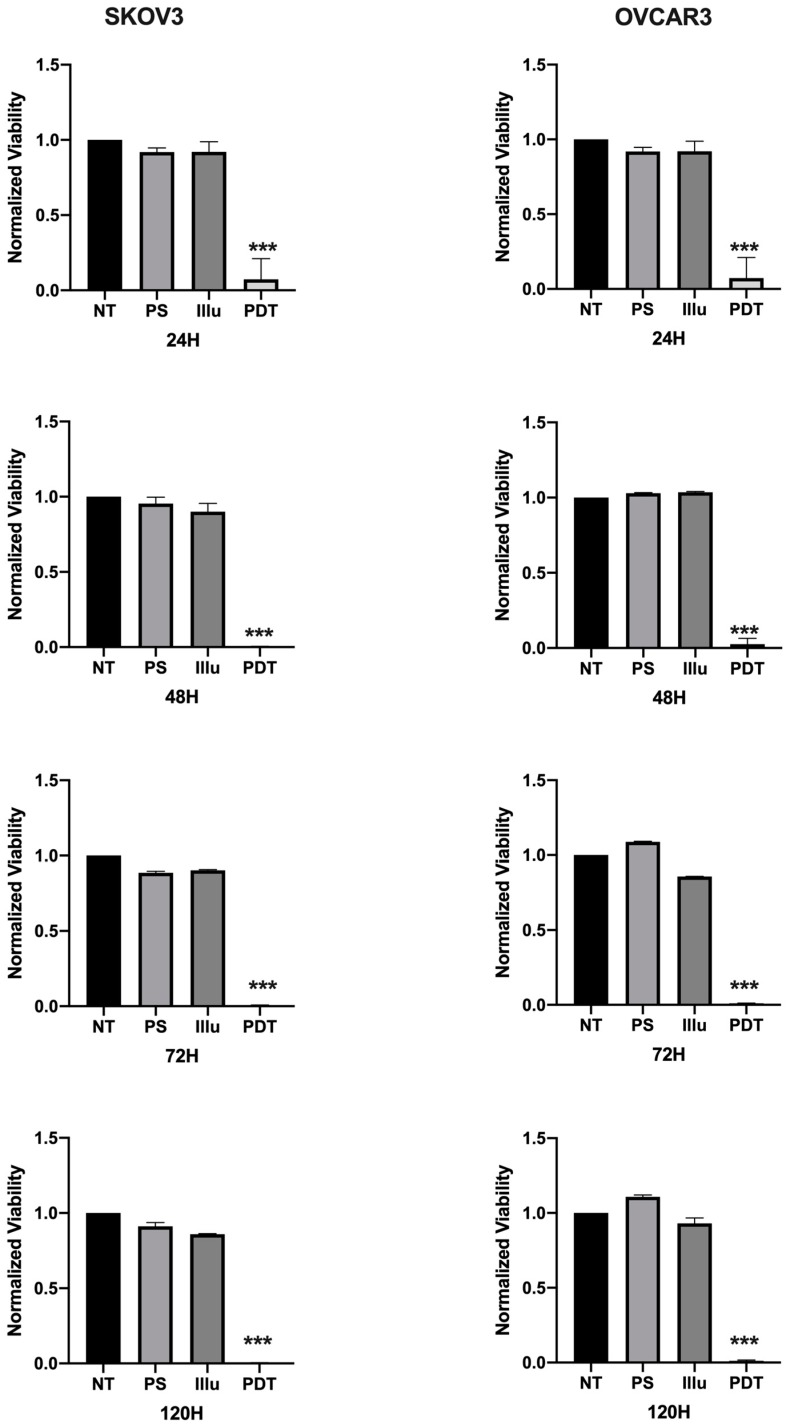
Percentage of Viability for OVCAR3 and SKOV3 at 24 h, 48 h, 72 h, and 120 h post-illumination. NT: non-treated, PS: Photosensitizer only, ill: illumination only, PDT: illumination in the presence of PS. Results are presented as means of three independent experiments, expressed in % of the NT. Rank-sum Mann–Whitney statistical test was performed, all quoted *p*-values are two-sided, with *p* ≤ 0.0001 (***) being considered statistically significant for the first and highly significant for the others. *n* = 3.

The impact of the PDT treatment was observable, looking at the morphological aspect of cells, after only 24 h of treatment. Indeed, cells subjected to PDT seem to lose cell-to-cell junctions as well as cell-to-surface adhesion. Furthermore, cells were floating in the culture medium. In fact, 24 h post-PDT, cells had detached and shrunk with different debris formations (>10 µm). This is even more interesting, as none of these changes were observed under the other control conditions (Figure 3). Regarding the viability and metabolism, the untreated OVCAR3 cells displayed high viability, which increased over time. For cells brought into contact with PS and those treated only with light, a slight decrease can be noted; however, this difference was not statistically significant. Furthermore, 24 h post-illumination, this decrease was more significant and sustained throughout the assay (until 120 h post-PDT). A similar result was found with SKOV3 cells, the only difference being that, for cells subject to PS, a slight (but not significant) increase in viability was observed (Figure 4).

### 3.2. Impact on the Human PBMC of the OVCAR3 and SKOV3 Secretome after PDT

#### 3.2.1. Evaluation of the Tumoral Secretome on the Viability and Proliferation of Human PBMC

**Figure 5:**
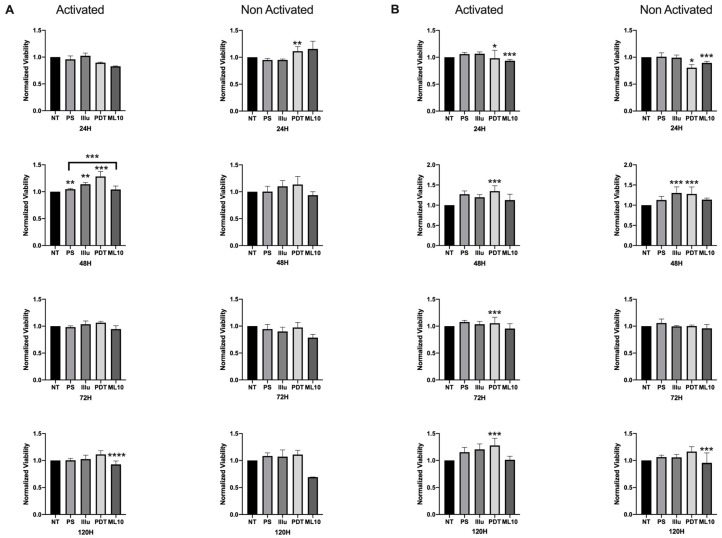
Percentage of Viability test for activated and non-activated PBMC in the presence of OVCAR3 and SKOV3 secretoma: (**A**) OVCAR3 or (**B**) SKOV3 cells 24 h, 48 h, 72 h, and 120 h post-culture. NT: non-treated, PS: Photosensitizer only, illu: illumination only, PDT: illumination in the presence of PS, ML10: the raw culture media of PBMC. Results are presented as means of three independent experiments expressed in % of the NT. Rank-sum Mann–Whitney statistical test was performed to compare data to NT control (or other, where stated). All quoted p-values are two-sided, with *p* ≤ 0.05 (*), *p* ≤ 0.001 (**), *p* ≤ 0.0001 (***) and *p* ≤ 0.00001 (****), being considered statistically significant for the first and highly significant for the others. *n* = 3.

When the PBMC were activated and cultured in the presence of 50% of the media of SKOV3 and OVCAR3 cell cultures, we noted a more important viability at 48 h, compared to the non-activated PBMC. This difference in the relative viability was sustained over time, until 120 h of incubation. PDT treatment operated by increasing the viability of activated PBMC, particularly at 48 h and 120 h (OVCAR3, Figure 5A) and at each time point (SKOV3, Figure 5B), where this increase was statistically significant. On the contrary, when the PBMC were not activated, no difference of viability rate was detected for OVCAR3 treated by PDT. For SKOV3, significant events can be noted—notably, a slight decrease at 24 h and an increase at 48 h—but as it was the same increase for light-only treated cells, the rationality of this increase is debatable.

**Figure 6:**
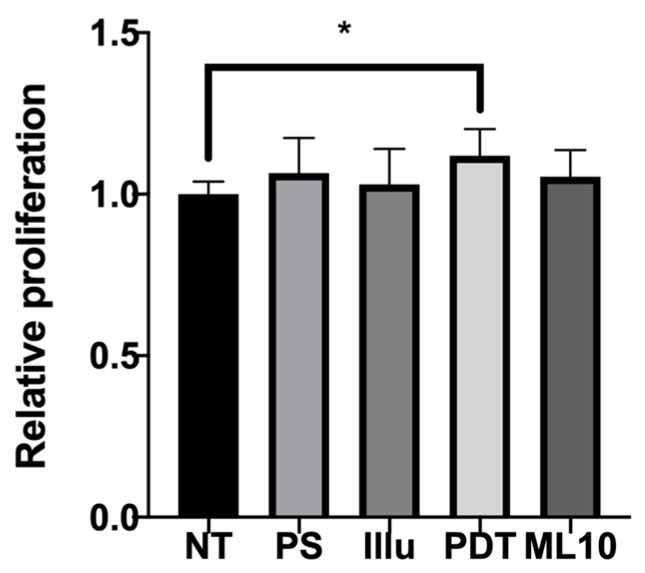
Proliferation assay of activated human PBMC culture with conditioned media of five conditions at 120 h NT: non-treated, PS: Photosensitizer only, illu: illumination only, PDT: illumination in the presence of PS, ML10: the raw culture media of PBMC. Results are presented as means of three independent experiments expressed in relative value of the NT. Ordinary One way Anova statistical test was performed, all quoted p-values are two-sided, with *p* ≤ 0.05 (*) being considered statistically significant. *n* = 3.

To assess the results of viability assays, we decided to check the proliferation rate upon 120 h of incubation. The proliferation rate of PBMC was still boosted at 120 h of incubation in the presence of OVCAR3-conditioned media (Figure 6). Indeed, compared to the non-treated tumor cell supernatant or even the raw culture media, the PDT conditioned media was still inducing a significant increase of proliferation.

**Figure 7:**
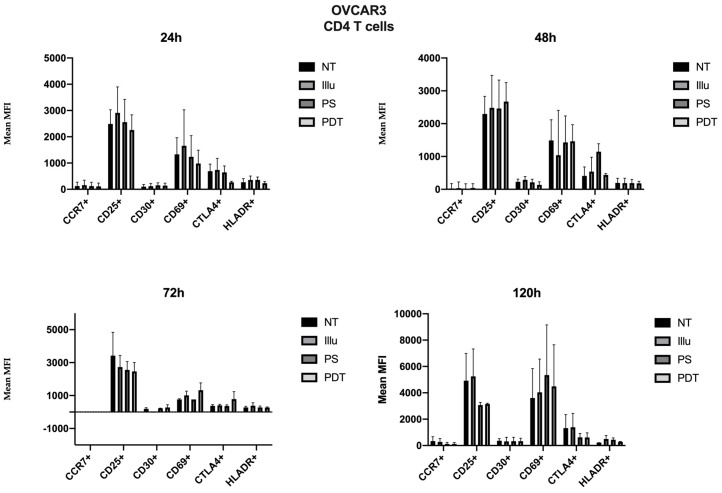
FACS analysis of activating markers of CD4+ T cells cultured with conditioned media of OVCAR3 cells at 24 h, 48 h, 72 h and 120 h. NT: non-treated, PS: Photosensitizer only, illu: illumination only, PDT: illumination in the presence of PS, ML10: the raw culture media of PBMC. Results are expressed in Mean of MFI (*n* = 3 independent donors).

**Figure 8:**
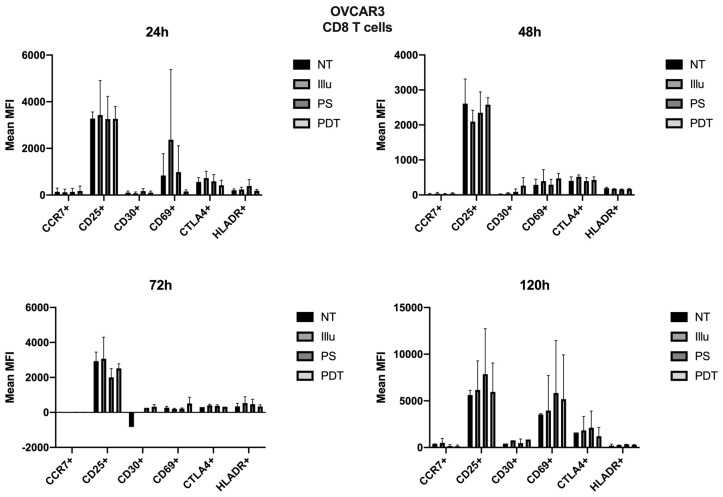
FACS analysis of activating markers of CD8+T cells cultured with conditioned media of OVCAR3 cells at 24 h, 48 h, 72 h, and 120 h. NT: non-treated, PS: Photosensitizer only, illu: illumination only, PDT: illumination in the presence of PS, ML10: the raw culture media of PBMC. Results are expressed in Mean of MFI (*n* = 3 independent donors).

As the immunomonitoring performed on PBMC was very clear and showed no differences in terms of the prevalence of immune populations (T, B, monocyte, NK, DC, Treg cells, and so on), we then chose to focus on the analysis of lymphocyte activation. Thus, we investigated the impact of conditioned media of ovarian cancer cells subjected to PDT on PBMC by studying the activation status of various T lymphocyte populations, including CD4 + and CD8 + T cells. Regarding the effect of the supernatant on OVCAR3 cells, it can be observed that the addition of PS and a fortiori PDT favored a slight increase in the CD30 late activation marker for CD4+ T cells associated with a decrease in CTLA4 (see Figure 7). For CD8+ T cells, there was a slight increase in the CCR7 associated with a decrease in the CD69 (see Figure 8). At 48 h, it can be noted that the expression of the CD25 was predominant; however, there was no difference between each treatment. It can still be noted that CD4+ T cells overexpressed slightly more CD25 following PDT, with a slight decrease in CD30 (see Figure 8). Meanwhile, TCD8+ saw a significant increase in the CD30 associated with a slight increase in CD69 (see Figure 8). Activation affected all lymphocytes, regardless of treatment. However, the PDT had a differential influence and a new wave of activation can be noted with the presence of CD25 for both CD4+ and CD8+ T cells (see Figure 7 and Figure 8). We see, overall, that the early activation of CD8+ T cells at 24 h was maintained, as CD30 was then overexpressed. It is also worth noting the slight increase in CD69 on CD8+ T lymphocytes, which again suggests a new wave of activation (see Figure 8). At 72 h, we can observe overexpression of CD69 for both CD4+ and CD8+ T lymphocytes. This clear increase confirms the wave of activation which began at 48 h. However, although it remained strongly overexpressed, there was a trend towards reduction in CD25 expression for both types of T cells (see Figure 7 and Figure 8). Finally, at 120 h, only CD8+ T cells still seemed able to respond, as the activation wave continued with overexpression of CD69 and a slight increase in CD30 expression. Meanwhile, for CD4+ T cells, the decrease in CD25 expression persisted and the other markers were no longer noticeable (see Figure 7 and Figure 8).

**Figure 9:**
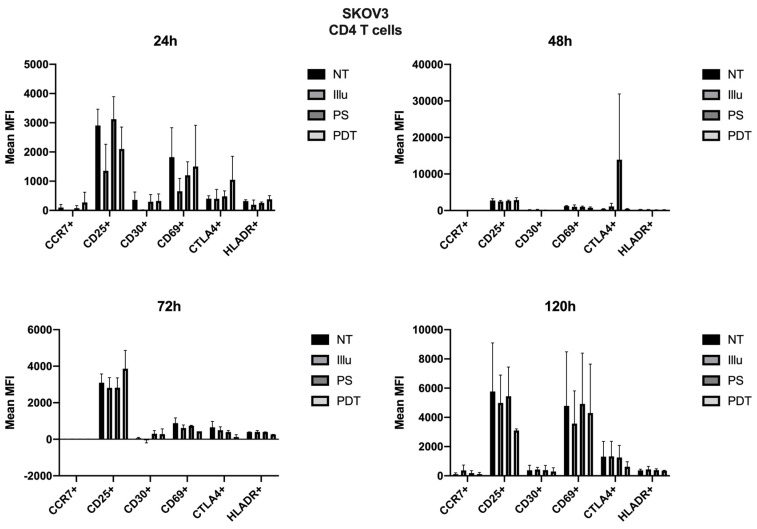
FACS analysis of activating markers of CD4+T cells cultured with conditioned media of SKOV3 cells at 24 h, 48 h, 72 h, and 120 h. NT: non-treated, PS: Photosensitizer only, illu: illumination only, PDT: illumination in the presence of PS, ML10: the raw culture media of PBMC. Results are expressed in Mean of MFI (*n* = 3 independent donors).

**Figure 10:**
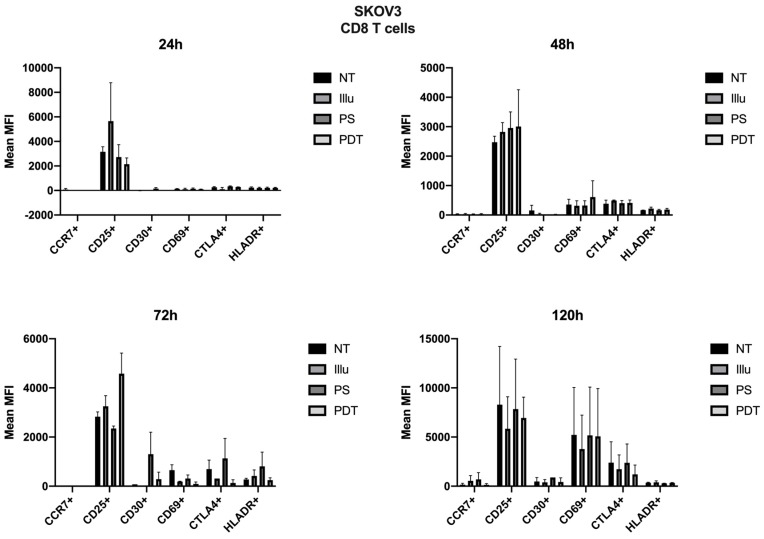
FACS analysis of activating markers of CD8+T cells cultured with conditioned media of SKOV3 cells at 24 h, 48 h, 72 h, and 120 h. NT: non-treated, PS: Photosensitizer only, illu: illumination only, PDT: illumination in the presence of PS, ML10: the raw culture media of PBMC. Results are expressed in Mean of MFI (*n* = 3 independent donors).

For SKOV3, the profile was quite different. Therefore, we can note cellular activation of the treated cells, notably with the overexpression of CD25; however, this was in a general and, therefore, non-specific way. At 72 h, there was a new wave of activation of CD4+ T cells. In fact, in addition to a good expression level of CD25, we observed an increase in CD69 and a decrease in CTLA4 expressions (see Figure 9). For TCD8+, CD25 was well-expressed for cells treated with PDT, but CD69 tended to have its expression reduced (see Figure 10). Finally, at 120 h, even if the CD25 and CD69 markers had a relatively more important expression than the others, they still tended to decrease. However, comparatively to the different conditions, this had no specificity. All markers seemed to decrease in expression.

#### 3.2.2. Evaluation of the Impact of EVs Produced by Cancer Cells on Human Immune Cells

**Figure 11:**
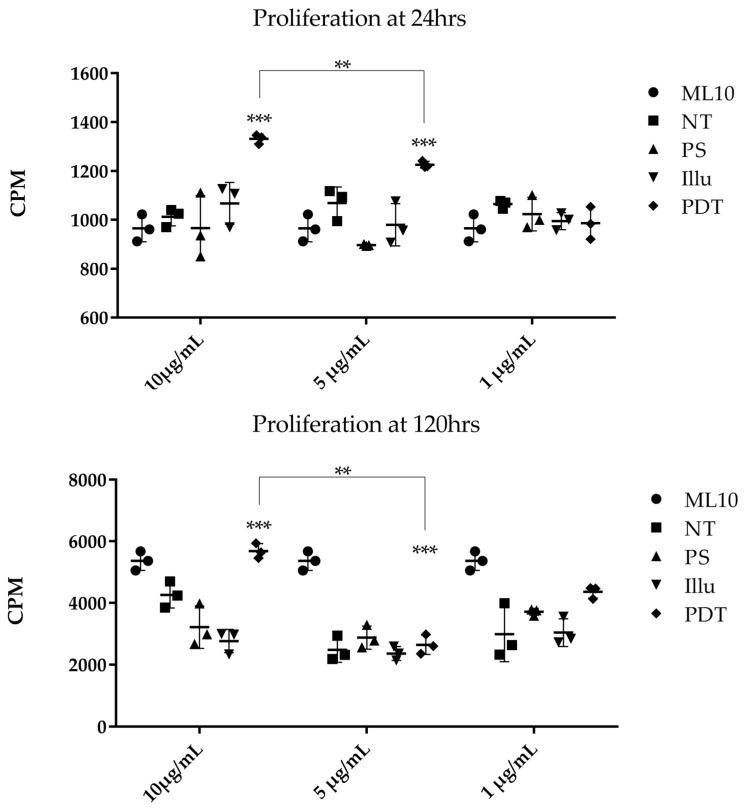
Proliferation assay of activated human PBMC cultured with different doses of EVs isolated from different SKOV3 culture medium at 24 h and 120 h. EVs were isolated from the supernatant of SKOV3, either non-treated (NT), subjected to the Photosensitizer (PS) or light (illu) only, or PDT treated (PDT), and compared to PBMC cultivated in their normal culture media (ML10). Results are presented as means of three independent experiments expressed in CPM. Two-way ANOVA test was performed. All quoted p-values are two-sided, with *p* ≤ 0.001 (**), *p* ≤ 0.0001 (***) being considered statistically significant for the first and highly significant for the others. *n* = 3.

We evaluated the impact of EVs isolated from the supernatant of ovarian tumor cells subjected to PDT or its components on the proliferation capacity of human PBMC. Activated PBMC were cultured at different time points with variable concentrations of EVs isolated from the supernatant of treated SKOV3 tumor cells (Figure 11). At 24 h post-culture, we observed a significantly higher cellular proliferation in the presence of EVs (10 µg/mL and 5 µg/mL) isolated from the PDT-treated condition, compared to the other ones. This effect was not observed when using 1 µg/mL of the same EVs.

At 120 h, the proliferation statistically increased when PBMC were cultured with 10 µg/mL of EVs from PDT-treated SKOV3 and a significant decrease was noted at 1 µg/mL. Whether at 24 h or 120 h, it seems that the growth increase was dependent of the concentration of EVs. In this sense, a statistically significant difference can be noted between the 10 vs. 5 µg/mL condition at 24 h and vs. 1 µg/mL at 120 h, respectively. On the other hand, no difference in the cellular proliferation was described when the PBMC were not activated and cultured in the same way with EVs from SKOV3-treated supernatant at 120 h. Even if the SKOV3 supernatant seemed to slightly increase their proliferation, it was not a significant effect.

#### 3.2.3. Evaluation of the Cytokines in the Secretome of Treated Ovarian Cancer Cells

**Figure 12:**
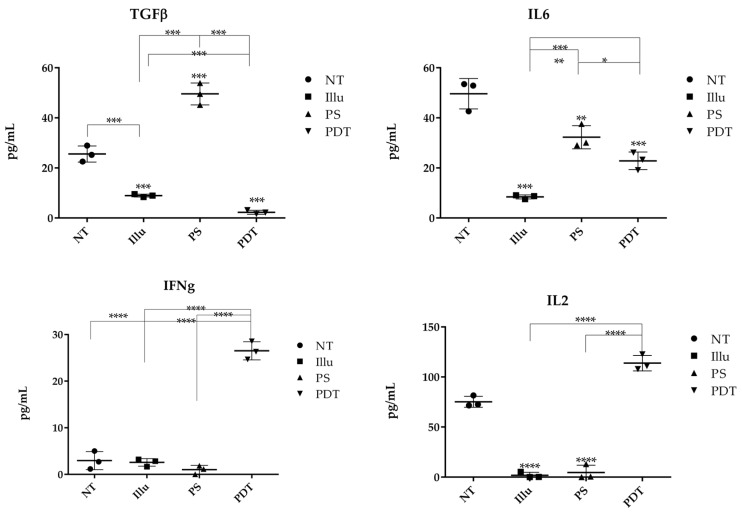
ELISA of different cytokines present in the medium culture of OVCAR3 cells under different conditions NT: non-treated, PS: Photosensitizer only, ill: illumination only, PDT: illumination in the presence of PS, ML10: the raw culture media of PBMC. Results are presented as means of three independent experiments expressed in pg/mL. Two-way ANOVA test was performed. All quoted p-values are two-sided, with *p* ≤ 0.05 (*), *p* ≤ 0.001 (**), *p* ≤ 0.0001 (***), and *p* ≤ 0.00001 (****) being considered statistically significant for the first and highly significant for the others. *n* = 3.

We performed ELISA on the supernatant of tumor cells of ovarian carcinoma treated or not with PDT or its components to determine whether tumor cells can release cytokines after treatment (Figure 12).

The untreated cancer cells produced TGFβ at a concentration of 28.94 pg/mL. However, when illuminated, there was a significant decrease in cytokine concentration (8.95 μg/mL). Conversely, when the cells were in contact with PS, there was a significant increase in cytokine concentration (49.56 pg/mL). However, after PDT or even illumination only, there was a significant decrease in TGFβ (1.36 pg/mL and 3.96 pg/mL, respectively). Consequently, the results obtained suggest that PDT decreases the production of the immunosuppressive cytokine TGFβ by ovarian carcinoma cells. Concerning IL6, the results indicate that, for untreated OVCAR3 cells, there was a natural IL6 secretion at a concentration of 53.45 μg/mL, which is a significant amount. For illuminated OVCAR3 cells or for OVCAR3 cultured with PS, there was a sharp and significant decrease (9.06 μg/mL and 36.30 pg/mL, respectively) compared to untreated cells. Moreover, for the cells subjected to PDT, we noticed an important and significant decrease (19.12 pg/mL). We then measured the production of the cytokine IL2. According to our ELISA results, OVCAR3 cells were able to secrete IL2 at a concentration of 72.50 pg/mL. On the other hand, for the conditions of illumination or PS only, we observed a relative absence of IL2, with a concentration equal to 0.96 pg/mL or 5.60 pg/mL, respectively. In contrast, when the cells were subjected to PDT, there was a significant increase in IL2 production, which almost doubled (122.50 μg/mL). PDT seems to favor the secretion of IL2. Regarding the release of IFNγ, when the cells were not treated, the concentration of IFNγ was very low (2.72 μg/mL). After illumination or with the PS, we observed a slight decrease to 3.20 pg/mL or 2.85 pg/mL of IFNγ, respectively. Finally, when the cells were subjected to PDT, a large increase (tenfold the cytokine production) was observed (28.55 μg/mL). These results suggest that PDT is likely to promote the production of IFNγ by ovarian carcinoma tumor cells.

## 4. Discussion

The aim of this study was to evaluate the impact of PDT on ovarian cancer cells and to analyze the consequences of this treatment on the regulation of the human immune system. First, we evaluated the efficacy of a new folate-conjugated photosensitizer on ovarian tumor cells, and then studied the impact of the secretome of ovarian tumor cells subjected to PDT on PBMC.

In order to validate the efficacy of the new folic acid-coupled photosensitizer, we first verified the expression of the folate receptor, both in ovarian tumor cell lines and in human primary immune cells, by RT-QPCR and flow cytometry. Transcriptomic analysis indicates that no expression of FOLR1 was found in immune cells. Moreover, FOLR2 expression was not detected in ovarian tumor cancer cells [25,26]. Furthermore, flow cytometry analysis of the FOLR1 protein expression was correlated with the Q-PCR results, confirming the higher expression of FOLR1 in OVCAR3 cell lines. Thus, the presence of folate receptors on both tumor cell lines suggests that they are both potentially sensitive to the new-patented photosensitizer. 

To confirm the effect of PDT using this photosensitizer on ovarian tumor cells, we first evaluated cell death via changes in their morphological appearance by photonic microscopy. The results exhibited clearly that the OVCAR3 and SKOV3 ovarian cancer cell lines were sensitive to PDT, hence validating the effectiveness of the new PS. These results were correlated with a viability test on SKOV3 and OVCAR3 tumor cells subjected to PDT. We noticed that SKOV3 and OVCAR3 subjected to PDT presented a massive and significant decrease in their cell viability over time. On the other hand, there were no notable changes in the viability of tumor cells treated with only one component of the treatment. These results confirm the effectiveness of the PS and show that PDT is capable of inducing the death of ovarian tumor cells with a very rapid effect, as 90% of tumor cells died after only 1 h of illumination.

Other folate-coupled pyropheophorbide conjugates have been previously developed [27]. However, as far as we know, no PS has shown the same results on intraperitoneal ovarian cancer cells. Compared to other photosensitizers, this new PS has the capacity to induce higher cellular death 24 h after illumination [28]. Furthermore, most of the FR-targeted PS are coupled with nanoparticles; however, previous observations have suggested that the application of NPs for intra-peritoneal delivery is limited by their rapid clearance from the peritoneal cavity due to lymphatic drainage [29]. Therefore, we bypassed this problem with a folate chemically coupled PS. Therefore, compared to existing photodynamic therapies for ovarian cancer [30], our study used a photosensitizer that could specifically target the ovarian cancer cells. In fact, most of the PDT studies have been conducted using 5-aminolevulinic acid methyl ester hydrochloride (5-methyl-Ala), a precursor of protoporphyrin IX (PpIX) in the heme metabolic pathway [31]. This popular precursor has been already tested on five ovarian cancer cell lines (3 serous tumor, 1 clear-cell, and 1 mucinous tumor). Although porphyrin and heme biosynthesis are well-understood mechanisms, the mechanism of PpIX accumulation in cancer cells after 5-methyl-Ala administration remains unclear [32].

In a second step, we studied the impact of the PDT-treated ovarian tumor cell’s secretome on human peripheral blood mononuclear cells. In fact, as mentioned in the introduction, the effectiveness of cancer treatment is highlighted by its capacity to induce immunosurveillance by maintaining an anti-tumor immune response. Considering that PBMC are the key effectors of immune response, we evaluated the capacity of PDT to induce such immunosurveillance, by culturing conditioned supernatants of the SKOV3 and OVCAR3 tumor cells with human PBMC. These PBMC were then subjected to viability tests at 24 h, 48 h, 72 h, and 120 h post-treatment, along with a proliferation assay at the last time point.

PBMC cultured with OVCAR3 or SKOV3 supernatant subjected to PDT showed an increase in viability after 1 h of culture. This reproducible increase in mitochondrial metabolism was sustained through time until 120 h of incubation. In the same way, we observed that the proliferation of treated PBMC was also statistically increased after 120 h of culture.

Thus, these results suggest that PDT is likely to modify the secretome of tumor cells, in favor of the activation of the mitochondrial metabolism of immune cells. This hypothesis was also supported by the increase of the proliferation rate of PBMC cultured with PDT-conditioned media. Considering these results, one can imagine that there are, in the secretome of PDT-treated ovarian tumor cells, some factors that may be responsible for immune system activation. These factors should be subjected to proteomic analyses; however, our hypothesis is that it may only be partly linked to the EVs present in the secretome of the ovarian tumor cells or the ability of treated tumor cells to secrete effector cytokines.

Moreover, the presence and increase of late activation markers on T lymphocytes suggests that T lymphocytes, whether CD4 or CD8, were already in the process of activation. Concerning CD8+ T lymphocytes, the presence of CCR7 also indicated that CD8+ effector T cells would tend to migrate to the draining lymph nodes. These results suggest that the supernatants of OVCAR3 submitted to the PDT promote activation. It is a very early process as, at 24 h, we already see signs of T lymphocyte activation. Interestingly, PDT appears to promote immunoactivation, with a continued activation of CD8+ T cells and several waves of activation over up to five days. For SKOV3, the profile was quite different; CD4+ T cells appeared to be much more sensitive, as there were increases in the CCR7 and CTLA4 markers. These results suggest that early activation of CD4+ T lymphocytes could have occurred at first contact with the SKOV3 supernatants treated with PDT at 48 h. While nothing remarkable happened for TCD4+, there was a clear increase in CD25 and CD69 expression for TCD8+. This indicates early activation of cells newly sensitized to PDT products. In conclusion, a different profile was observed, compared to OVCAR3 cells. Indeed, although both supernatants activated the cells, the sensitivity of T lymphocytes to the products of SKOV3 cell supernatants subjected to PDT was more pronounced. What is interesting is that the cells seem to have reproduced the pattern of activation of the TH1 cell response in a co-ordinated manner. It should be noted that it was, first of all, the CD4+ T lymphocytes that seemed to have been more sensitized, followed by a very clear response from the TCD8+. These results are very interesting, considering that activation markers are an essential element in understanding the impact of PDT on the immune response. In addition to proliferation, which signals the first stages of activation, the fate of cells and their potentiality are revealed in this temporal monitoring of cell evolution. However, these in vitro results will have to be confirmed by extensive in vivo studies in adequate mouse models, but especially in humans in clinical trials.

EVs are mostly of endocytic origin and play an important role in several mechanisms, such as cancer biology. They can be responsible for cell-to-cell communication by the transfer of proteins, nucleic acids, and lipids [33,34]. In ovarian cancer, these EVs are believed to promote peritoneal dissemination, especially by favoring the interaction between cancer cells and their microenvironments [35,36]. Several new studies have suggested that, due to their immunomodulatory capacities, EVs are major potential players in cancer immunotherapy [37]. This is essentially due to the role of DC-derived EVs capable of activating T-cells and NK cells [38]. EVs can, therefore, be considered as real therapeutic targets, especially in ovarian cancer where EVs are found in large quantities in patient ascites. To verify all of these hypotheses, we studied the impact of EVs derived from the secretome of ovarian tumor cells subjected to PDT on PBMC. For this purpose, the EVs of the supernatants of the SKOV3 tumor cells, cultured according to the different conditions, were brought into contact in vitro with healthy human PBMC. These PBMC were then subjected to a proliferation test with different EV concentrations. We observed that the proliferation of PBMC was different, according to the concentration of co-cultured EVs. In fact, there was a dose dependent activation when the PBMC are activated. Specifically, the higher the EV concentration, the greater the proliferation of PBMC. This activation was accentuated after 120 h of culture. No difference was observed when the PBMC were not activated. These results suggest that tumor EVs from ovarian tumor cells subjected to PDT are likely to activate the proliferation of immune cells and may induce a potential abscopal effect.

In order to elucidate the effect of PDT on immunity, we first tested the ability of PDT to modify the ability of ovarian tumor cells to secrete cytokines. Immunosuppressive (TGFβ), immunoactivating (IL2, IFNγ), and pro-inflammatory (IL6) cytokines were tested. IL6 is known to be a pro-inflammatory cytokine capable of promoting inflammatory processes [39]. It is well-known that, in the case of inflammation, IL6 cytokines have two major roles: one of them is a direct effect on innate immune cells and the second is indirect, via activation of local stromal tissue [40]. However, this could be problematic when it comes to cancer. In fact, inflammation promotes tumor progression and dissemination of metastases [41]. Moreover, it has been described that IL6 acts as a pro-metastatic factor in ovarian cancer cells [42,43]. Interestingly, our results show that, for untreated OVCAR3 cells, there were high concentrations of this cytokine. However, the cells subjected only to illumination or cultured with PS showed a decrease in this cytokine, compared to untreated cells. Moreover, when the cells were subjected to PDT, we also saw a decrease in the IL6 cytokine. Thus, our results suggest that OVCAR3 cells treated with PDT decrease their IL6 cytokine production. These first results are very encouraging, regarding the use of PDT as an immunoactivating adjuvant.

Then, we investigated the production of IL2, a proliferative and pro-survival cytokine able to interact with a variety of immune cells, but mainly on the lymphocytes which constitutively express low-affinity IL2 receptors. The overall physiological role for IL-2 is to meticulously co-ordinate the Th1-type immune response against certain pathogens and cancers [44]. It has been well-described that IL-2 plays a major role in the homeostasis of the immune response, favoring the survival and proliferation of T lymphocytes. According to our results, when the OVCAR3 were not treated, the supernatant of the untreated cancer cells presented a significant presence of IL2. On the other hand, for the conditions of illumination or PS only, we observed an absence of the IL2 cytokine production. In contrast, when the cells were subjected to PDT, there was a significant increase in IL2 production. Thus, these results show that PDT promotes the secretion of IL2 by ovarian carcinoma cells which, in turn, favors a proliferative effect in immune cells, particularly in T Lymphocytes. Indeed, activation based on anti-CD3 and anti-CD-28 stimulation mimics specific T cells activation. Here, the proliferation rate of PBMC stimulated in this way was increasing with the contact of the supernatant to PDT-treated ovarian cancer cells. This is all the more important, as lymphocytes are the most effective effectors in antitumor therapy; their activation, which is specific for antigens, can lead to long-term protection through the establishment of an immunological memory. Regarding the expression of IFNγ, after illumination, we observed a slight increase in this cytokine. On the other hand, when cultured with PS alone, we were not able to detect such an effect. The PS thus seems to block or inhibit IFNγ secretion. However, when the cells were subjected to PDT, an important increase in IFNγ was observed. These results suggest that PDT likely promotes the production of IFNγ by the ovarian carcinoma cells and, therefore, to participate in a potential immunoactivating effect. In fact, IFNγ can prevent the development of malignant tumors and metastases [45]. Particularly, IFNγ can inhibit angiogenesis, stimulate the maturation of B and T lymphocytes, and activate other types of immune cells such as monocytes [46]. In addition, its role in inhibiting immunosuppressive pathways and particularly the secretion of IL10 makes this cytokine a weapon of choice for the antitumor immune response. Finally, we tested the immunosuppressive cytokine TGFβ, which is capable of inhibiting the immune response. Compared to untreated cells, when cancer cells were illuminated, there was a decrease in the TGFβ concentration. However, when the cells were in contact only with PS, there was a significant increase in this cytokine concentration. After PDT, there was a significant decrease of the TGFβ concentration, suggesting that the treatment could directly block the immunosuppressive cytokine TGFβ secretion by ovarian carcinoma cells. This effect is beneficial, as it limits the immunosuppressive microenvironment favorable to tumor growth. In fact, tumor cells can develop mechanisms to enhance and use TGFβ-induced immunosuppressive effects [47]. Thereby, our results suggest that PDT seems to inhibit the tumor suppressor pathway orchestrated by TGFβ. To summarize our results concerning the secretion of cytokines by ovarian tumor cells and their behavior following PDT treatment, we can say that PDT seems to establish an environment favorable to an adaptive effective immune response. This is all the more important, as tumor cells usually create an environment strongly unfavorable to the development of an effective response. These results are also in line with the preliminary observations we obtained by cytometric analysis, in which we observed activation of the immune cell populations in contact with the supernatant of ovarian cancer cells treated with PDT. Indeed, a specific increase in CD8+ and CD4+ T lymphocytes was observed. This indicates the development of a TH1 antitumor immune response.

## 5. Conclusions

In conclusion, in the present study, we evaluated the effect of a new folate conjugated photosensitizer dedicated to intraperitoneal ovarian cancer treatment. We showed that this PS, upon illumination, could induce cell death of different ovarian tumor cells. Furthermore, PDT using this new PS seems to favor activation of the immune response by inducing the secretion of effective cytokines and inhibiting the pro-inflammatory and immunosuppressive ones, as well as releasing EVs which are prone to activating immune cells. Finally, we show that PDT can activate CD4+ and CD8+ T cells, resulting in a potential immunostimulating process. All the results of this pilot study therefore indicate that PDT treatment with this new PS may not only be effective in rapidly and directly destroying target tumor cells, but also promoting the development of a protective long-term antitumor immune response. Other points need to be evaluated and will certainly be addressed and clarified in future clinical trials with this new-patented photosensitizer. These data thus provide good prospects for the treatment of micrometastases of intraperitoneal ovarian carcinosis which are currently inoperable.

## Figures and Tables

**Table 1:** List of the Photophysical properties of the PS in ethanol.

Name	ε_Soret band_ (L.mol^−1^.cm^−1^)	λ_QI_ (nm)	ε_QI_ (L.mol^−1^.cm^−1^)	ϕ_F_ (± 0.02)	ϕ_Δ_ (± 0.05)	τ_F_ (± 0.1 ns)	τ_Δ_ (± 1 μs)
Pyro-PEG-FA	74 081	668	35 306	0.30	0.54	6.4	13

ε: molar extinction coefficient; Q: Q band; λ: wavelength; F_F_ fluorescence quantum yield; FD: singlet oxygen luminescence quantum yield; τf: fluorescence lifetime; tD: lifetime of singlet oxygen.

**Table 2:** List of the primers used for QPCR.

	Primers	
**FOLR1**	5′-AGGTGCCATCTCTCCACAGT	5′-GAGGACAAGTTGCATGAGCA
**FOLR2**	5′-CTGGCTCCTTGGCTGAGTTC	5′-GCCCAGCCTGGTTATCCA
**18S**	5′-TCAAGAACGAAAGTCGGAGG	5′-GGACATCTAAGGGCATCACA
**GAPDH**	5′-GCCAAGGTCATCCATGACAACTTTGG	5′-GCCTGCTTCACCACCTTCTTGATGTC
**HPRT**	5′-CCCTGGCGTCGTGATTAG	5′-ATGGCCTCCCATCTCCTT

**Table 3:** List of the antibody panel used for flow cytometry.

Panel	Antibodies	Isotypic Controls
Activation	CD30-APC-Vio770	Mouse IgG1-APC-Vio770
	CD69-PE-Vio770	Mouse IgG1-PE-Vio770
	Anto-HLA-DR-PerCP-Vio700	Mouse IgG2a-PerCP-Vio700
	CD152-APC	Mouse IgG2a-APC
	CD197	REA Control (S)-PE
	CD25-VioBright FITC	Mouse IgG2b-VioBright FITC
Population	CD8-VioGreen	Mouse IgG2a-VioGreen
	CD4 (VIT4)-VioBlue	Mouse IgG2a-VioBlue
